# Mortuary rites of the South Fore and kuru

**DOI:** 10.1098/rstb.2008.0074

**Published:** 2008-11-27

**Authors:** Jerome T. Whitfield, Wandagi H. Pako, John Collinge, Michael P. Alpers

**Affiliations:** 1 MRC Prion Unit and Department of Neurodegenerative Disease, UCL Institute of Neurology, National Hospital for Neurology and NeurosurgeryQueen Square, London WC1N 3BG, UK; 2 Centre for International Health, ABCRC, Shenton Park Campus, Curtin UniversityGPO Box U1987, Perth, WA 6845, Australia; 3 Papua New Guinea Institute of Medical ResearchPO Box 60, Goroka, EHP 441, Papua New Guinea

**Keywords:** Fore, mortuary rites, kuru

## Abstract

This paper is part of a wider study to explain the historical spread and changing epidemiological patterns of kuru by analysing factors that affect the transmission of kuru. Part of the study has been to look at the mortuary feasts that were the means of transmission of the kuru agent. This paper shows the complexity of Fore eschatology, and the variations and contradictions of human behaviour in relation to mortuary rites and the transmission of kuru. It also confirms that oral ingestion was the primary route of inoculation though some cases of parenteral inoculation may have occurred. The exclusion of alternative routes of transmission is of importance owing to the dietary exposure of the UK and other populations to bovine spongiform encephalopathy prions.

## 1. Introduction

Early anthropological studies in the kuru-affected region suggested that the reason for the practice of eating dead kinfolk was primarily gastronomic, and that it had no ritual or spiritual role despite the normative rules about who should or could consume various parts of the body at mortuary feasts (Berndt 1962; Glasse 1963, 1967).

Later works have explained the role of endocannibalism in the epidemiology of kuru and emphasized that the body was eaten out of love as well as for gastronomic appreciation, which was not the intended purpose of the practice but its result. They also pointed out the importance of kinship, gender and age in the participation at mortuary feasts to the transmission and spread of the disease (Alpers 1968; Mathews *et al*. 1968; Lindenbaum 1979; Klitzman *et al*. 1984).

More recently, the term transumption has been used to describe the eating of dead kinfolk among the Fore. Transumption has been defined as:

…the mortuary practice of consumption of the dead and incorporation of the body of the dead person into the bodies of living relatives, thus helping to free the spirit of the dead; this practice had deep significance for the Fore people and their neighbours.

(Alpers 2007, p. 14)

Early researchers were quick to note that kuru was predominantly a disease of women and children. Though some descriptions of how the body was distributed at feasts were obtained, the information on variations of the mortuary practices throughout the kuru-affected region was limited (Glasse 1963, 1967). Some descriptions of mortuary practices emphasized the belief that brain tissue was purposely rubbed on the body of the mourners to support the idea that parenteral inoculation was the primary means of transmission of kuru (Gajdusek 1971).

The principal conclusions of this paper are that transumption had deep significance for the Fore people, that for eschatological reasons the body was totally consumed and that intentional rubbing of brain tissue on the mourners' bodies did not occur.

## 2. Fore cosmology

The land is called *bagina* in the Fore language; it is alive and created today's landscape. Once the *bagina* had finished creating the landscape, it created the *amani*, who are the guardians of the clans; the Fore are the descendants of their clans' guardians. As the human population increased, the *amani* withdrew to their own lands that became the sacred grounds of each clan, also known as *amani*. A clan's *amani* consisted of a mountain, lake, pine and palm grove, and a cave that led to *kwelanandamundi*—the land of the dead in the Fore cosmology. After a person's death, the souls of the person travel across the land bidding farewell to the *bagina* and the clan's *amani*, before continuing their journey to *kwelanandamundi*, the home of the ancestors.

## 3. The five souls of the Fore

A person has five souls (*auma*, *ama*, *kwela*, *aona* and *yesegi*) that depart from the body on the last breath but only the *auma* departs immediately to the land of the dead. The *auma* is similar to the western concept of a soul and consists of a person's good qualities. Once the *auma* has been forgiven by a close male affine for any transgressions committed during the deceased's life, it travels across the land saying farewell to the *bagina*. When it reaches the *amani*, it explains to the clan guardian why it has died and the *amani* feeds the *auma* and directs it on the road to *kwelanandamundi*. Food and water left at the sepulture by the family provide sustenance for the *auma* on its journey. Eventually it reaches a red river at the entrance to the land of the dead and having crossed this it is welcomed by the ancestors. The *auma* then has to wait for the *ama* (bones) and *kwela* (flesh) to arrive before it can be reborn as an ancestor.

The *ama* is a simulacrum of the *auma*, but more powerful, and remains in the land of the living until all the obsequies have been carried out correctly. It assists the family to complete the obsequies and avenge the death of the deceased if enemies are held responsible. When the deceased's body was eaten out of love and grief, the *ama* would give blessings to those who ate and the blessings increased their *aona*. After the mortuary rites have been completed, the *ama* departs to *kwelanandamundi*, from where it continues to assist the family when requested.

*Aona* is the deceased person's abilities and remains with the *ama* until it is passed on to the favourite child of the deceased, normally at the *agona* ritual that marks the end of the obsequies. All adults have an *aona* that consists of a whole range of abilities covered by one term. For example, a person who has an *aona* that enables him to share food at feasts might also never be short of food. In this case his *aona* might also make him a good hunter, an exceptional raiser of pigs or a successful gardener.

The *kwela* is the pollution from the decomposition of the flesh and blood of the body. The *kwela* travels on the wind as a cloud of pollution in the form of a man and harms the family members if the obsequies are not conducted properly. If a body was placed on a platform or in a basket, it had to be far from human habitation as the *kwela* would become immensely powerful as the body decomposed. If the body was buried, the *kwela* was less dangerous as the pollution was contained in the burial pit. If the body was eaten, the *kwela* remained in the wombs of the female affines, called *anagra* in Fore, during the period of decomposition to contain its dangerous effects, as it would not harm people with the same blood. It was during the *aluana* purification rite that the *kwela* was removed from the bodies of the *anagra* to the sepulture, which was the fireplace where the body had been cooked, and the *kwela* finally departed to *kwelanandamundi* with the *ama* after the *agona* rite had been performed.

*Yesegi* is found on the skin of the living and stays with the *kwela* until it is passed on to one of the children of the deceased before the *kwela* departs to *kwelanandamundi*. *Yesegi* is a person's occult power that in the past made him a great warrior and powerful sorcerer.

By performing the obsequies with the right motivation, the family ensures that the souls are recycled within the family or become an ancestor, all of which will assist the living. Although in the past there were different methods of disposing of the body among the Fore, the end purpose was the same in ensuring that the souls went to their respective destinations.

## 4. Methods of disposal of the dead among the Fore

In the Fore region bodies could be disposed of by burial; by placing them in a basket, or on a platform, in a bamboo or yellow sugar cane grove; or by transumption. In the South Fore, transumption was by far the most common method and bodies placed in sepultures were often removed and eaten out of love for the deceased.

The dying person would normally express their wishes as to how their body was to be disposed of; otherwise the family would decide. In the kuru-affected region, all methods of disposal of the body involved being eaten. If the body was buried it was eaten by worms; if it was placed on a platform it was eaten by maggots; the Fore believed it was much better that the body was eaten by people who loved the deceased than by worms and insects. By eating the dead, they were able to show their love and to express their grief. The ritual allowed the *aona* and *yesegi* to be recycled within the family and for the loved ones to receive blessings from the *ama*, which would strengthen their *aona*. The eating removed doubts about family or community loyalties as the *kwela* would attack any woman who ate whose family had been involved in the death of the deceased. By the eating of the body, the danger of the *kwela* during the period of what would have been decomposition was averted as the *kwela* was confined inside the *anagra* who ate the body, thus protecting the family. By performing the obsequies correctly, the relatives ensured that the souls of the deceased departed to *kwelanandamundi* and the deceased was reborn as an ancestor.

## 5. The practice of transumption

After death, the family and relatives mourned over the body of the deceased for 2–3 days and then, if it was to be eaten, it was taken on a stretcher to a bamboo, sugar cane or casuarina grove. These sites were chosen as they provided shade to the mourners and the deceased's souls, making them happy. Once the body was cut, the land welcomed and comforted the *ama* and *kwela* of the deceased.

The body was laid on a bed of edible greens on a tapa cloth placed on banana leaves. This was to ensure that, when the body was cut up, nothing was lost on the ground as this would have been disrespectful to the deceased and the *kwela* might harm the family members in anger. The body was divided among those *anagra* who had a right to it and the deceased's *anaso* (a woman's female guardian who lived in the same village as she married into, who would have a right to her body and her husband's when they died). The *anagra* would give the head of the deceased to the *anatu* (female agnates) when requested and sometimes the right arm. The senior *anagra* was in charge of cutting the body and the other elderly *anagra* assisted her as, being old, their *aona* was already waning, so the polluting effect from the body would not pose a danger to them or their elderly husbands. The women debarred the children from being at the place of the event, but the curious ones would hang around on the periphery of the activities in the hope of catching a glimpse of what was happening. Babies were kept 3–4 m away as the *kwela* might harm them. As the body was cut up, the pieces of meat were put in piles for each recipient on a breadfruit leaf on a banana leaf and the bones of the corresponding parts placed on top. When the torso was about to be cut open, the older women formed a wall around the body using their bark capes so that the younger women and children did not see the intestines and genitals when they were removed.

Once the body was divided up, the *anagra* cleaned their hands with banana skins, sugar cane or bamboo shoots. Fibres were used to clean the finger nails and the juice helped remove the blood; finally the women would wipe their hands on their grass skirts or bark capes. The cleaning materials were then burnt on the fire that was used to cook the body. The *anagra* and *anaso* took their piles of meat and proceeded to cut them into smaller pieces with the help of their daughters and daughters-in-law. They then shoved the strips of meat with wild ferns into bamboo containers and wiped their hands on wild ferns that were placed in the same bamboo cylinders. The tubes were then cooked on one fire and each *anagra* emptied half the content of her tubes on to a communal plate made of banana leaves. The meat on this pile was used to feed the *ename*, who were the women from nearby communities who came to share the grief of the family and affines. These women were fed pieces of meat directly into their mouths on the end of a sharpened stick, so they did not actually touch the meat. This was to protect the women from the *kwela* that might harm a woman if her husband had offended the deceased when he was alive. The women would chew *wesa* leaves on the way home to purify their mouths.

The *anagra* and *anaso* would eat their portions with their daughters, daughters-in-law and their children. The *anatu* (daughters-in-law, daughters and sisters of the deceased) and their children normally ate the head at the same time but occasionally it was eaten the following day. If the head was eaten on the same day, half the content of the bamboo tubes of the *anatu* was put on the communal pile for the *ename*. When the body was eaten, the *kwela* of the deceased entered the wombs of the *anagra* and remained there until *aluana* was performed. Among the Atigina (people who live in the central Fore region who speak *atikamana*), the widow received the genitals and intestines, which were not suitable to share with the *ename* so she shared these with other female relatives, but among the Pamusagina (people who inhabit the south of the Fore region) she received nothing and these parts were eaten by the *anagra*. During the night any remaining meat was taken to the widow's house and shared among the women who remained there during the night to share the grief of the widow and other family members. The bones were hung up in old bilums in the house and the head too if it had not already been eaten.

In the morning the women took any remaining meat, the bones and stones from a river bed to the fireplace that had been used the previous day to cook the body. They then performed an obsequy called *ikwaya ana* during which the rest of the body was eaten. The bones were dried by the fire so that they broke easily without sharp edges. Concave stones were placed on the ground containing a breadfruit leaf and a wild grass called *igagi*; the bones were placed on this with more *igagi* on the top and then crushed with another stone. This technique was used to ensure that none of the bone was lost during the process as it was important that the whole body was consumed. Once crushed, the bones and grass were placed in bamboo tubes, cooked and eaten. Finally, all the utensils used over the preceding 2 days were burnt on the fire. Sometimes the ashes from the bamboo utensils were mixed with wild green vegetables and eaten to ensure that the whole body was consumed. The exceptions were the jaw bone and collar bones, which were normally kept and worn by women in memory of the deceased and by men as a portal to request help from the *ama* of the deceased. In the evening, the women returned to the widow's house and continued to eat the body until it was all consumed.

Once the body had been eaten, a feast called *isosoana* (a feast for those who mourned) was prepared and the women in the widow's house were allowed out to take part in a purification ritual called *pepatakina*. The steaming leaves from the earth oven used to prepare *isosoana* were placed at the doorway of the hut and as the women left, they passed through the steam, which purified their bodies. The ritual allowed the women to move around in the community so that they could assist with the obsequies.

The following day the women caught rats in their gardens and the men hunted possums. When they had enough animals, the women performed a ritual in the widow's house called *aindu* during which the animals were put on the fire so that their fur and fat were burnt. It was believed that the smell would remove the *kwela* from the women. Afterwards, the carcases were rubbed on anything that the women who had partaken in the obsequies might have touched, such as doors or bilums, so that any remains of the deceased's body would be wiped onto the carcases, which were then cooked and eaten by the women. This was done to ensure that the whole body was eaten and the hamlet purified.

From the time the body was eaten, another obsequy started called *kavunda*, which means ‘eating wild greens’. This lasted for several weeks during which the relatives and friends would come and stay with the family to share their grief and bring them food. As most of the family's food had been used up to feed the guests, they now ate a poor diet of wild ferns, grasses and other wild plants. *Kavunda* was also a purification ritual as the wild vegetables were symbolic of the wild land of the dead called *kwelanandamundi* and informed the *kwela* and *ama* that they would soon be departing to the wild land of their ancestors. The widow was allowed to assist in collecting food, but had to remain in or at the back of her hut when she returned to the village. When grass started to grow on the sepulture, it symbolized that it was time for *kavunda* to end.

*Aluana* marked the end of *kavunda* and was a feast prepared with wild animals and vegetables. The men hunted possums and wild pigs, and the women and children caught garden rats and grubs. All the women who had eaten part of the body had a right to take part in this feast. During *aluana*, the *kwela* was removed from the wombs of the *anagra* and sent to the sepulture, which was the fire pit where the body was cooked.

*Igoghana* normally took place after *aluana* and the timing depended on when the family had enough pigs and vegetables. This feast was a compensation payment to the women for having eaten something that was not fit for human consumption. The names of men were called for this payment and the women received a share of the food.

The final obsequy was *agona* and this was a substantial payment that could take several years to prepare. Once this payment was made, the *kwela* and *ama* departed to *kwelanandamundi* taking the shadows (simulacrums that existed in the spirit world) of all the foods and goods that had been used in the obsequies. They said goodbye to the *bagina* and the *amani* and on their arrival in *kwelanandamundi* the deceased became a complete ancestor. The *kwela* and *ama* still continued to visit the widow and when she accepted a marriage proposal the future husband performed a ritual called *kilanau kasa* that freed the woman from her dead husband's *ama* and *kwela*. If she did not remarry, her dead husband's *ama* remained with her until she died.

The brain contained most of the infectious agent and its consumption was largely responsible for the transmission of kuru. The head of the deceased was placed over a fire to burn off the hair and then it was de-fleshed with a bamboo knife. A hole was made in the top of the skull using a stone and the brain was gradually removed by one of the older women whose hand would be wrapped in ferns. The tissue was then mixed with ferns and placed in bamboo tubes, normally two or three, and cooked. The head of a male or married female belonged to the affines but was nearly always given back to the family, and the head of an unmarried female belonged to the family. Children under the age of 3 were never fed any part of the body, but those aged between 3 and 6 were indulged even though they were not meant to eat the brain. It was believed that the brain tissue would stop children from growing properly so the children were not meant to eat it; however, this rule was enforced in some areas but not in others—indeed some participants held the belief that brain was good for the growth of young children. The parents were also worried that if a child squeezed the soft meat, some might fall on the ground and the *kwela* might harm the child for not showing respect to the deceased's body. The brain was considered a delicacy and the children would demand some from their mothers who naturally indulged them out of love. Males over the age 6 never consumed brain tissue, but females of all ages in the majority of South Fore communities consumed it. Sometimes the *anatu* would share half of the cooked brain with the *ename*. To ensure that the *kwela* departed to *kwelanandamundi* and the deceased became a complete ancestor, the entire body had to be eaten and, for this reason, the women ensured that the brain was all eaten. It was never rubbed on to their bodies, which would have been disrespectful of the dead.
